# Functional and Respiratory Capacity of Patients with Chronic Kidney Disease Undergoing Cycle Ergometer Training during Hemodialysis Sessions: A Randomized Clinical Trial

**DOI:** 10.1155/2019/7857824

**Published:** 2019-01-21

**Authors:** Antonio de Olival Fernandes, Yvoty Alves dos Santos Sens, Vivian Bertoni Xavier, Luiz Antonio Miorin, Vera Lúcia dos Santos Alves

**Affiliations:** ^1^MD, PT, Hospital Escola Vila Nova Cachoerinha, São Paulo, SP, Brazil; ^2^MD, PhD. Associate Professor in Santa Casa de Sao Paulo School of Medical Sciences, São Paulo, SP, Brazil; ^3^PT, PhD. Professor in Santa Casa de Sao Paulo School of Medical Sciences, São Paulo, SP, Brazil; ^4^PT, PhD. Associate Professor in Santa Casa de Sao Paulo School of Medical Sciences, São Paulo, SP, Brazil; ^5^University of Mogi das Cruzes. Mogi das Cruzes, SP, Brazil

## Abstract

**Purpose:**

Exercise is recommended for patients undergoing hemodialysis, to reduce the decrease in functional capacity secondary to the progression of chronic kidney disease. A cycle ergometer can be easily added to an exercise routine during hemodialysis sessions. The purpose of this article was to assess the results of a training protocol with the cycle ergometer during hemodialysis sessions on the respiratory function and functional capacity of patients with chronic kidney disease on hemodialysis.

**Method:**

In this randomized clinical trial (NCT no. 02834026), 39 patients undergoing hemodialysis were randomly allocated into two groups: the treatment group (TG, n = 20), who underwent a cycle ergometer protocol training, and the control group (GC, n = 19), not trained. The TG attended 24 training sessions, three times a week, during the intradialytic period. Training intensity was aimed at keeping the heart rate between 50 and 70% of its maximum. All participants were evaluated before and after the eight consecutive weeks of follow-up and had biochemicals data, anthropometric, functional, and respiratory outcomes evaluated.

**Results:**

A significant difference was observed between groups in forced vital capacity, forced expiratory volume in the first second, peak expiratory flow, maximal inspiratory and expiratory pressure, and Borg score and distance covered in the six-minute walk test. Improvement was also observed in biochemical and Kt/V test results for the TG.

**Conclusion:**

The systematic training regimen with a cycle ergometer resulted in benefits in the respiratory function and functional capacity in patients with chronic kidney disease undergoing hemodialysis.

## 1. Introduction

Patients with chronic kidney disease (CKD) may have poor physical conditioning, as reported in the literature. Deconditioning is hypothesized to be multifactorial and may be associated with the natural progression of the disease, malnutrition, anemia, myopathy, sedentary lifestyle, and muscular atrophy. The muscular impairment in these patients is evidenced by the decrease in strength and reduction in flexibility [1, 2].

Several studies have shown that the changes observed in the contractile capacity of the skeletal striated muscles also extend to the muscles of the rib cage [3, 4], which may, theoretically, lead to a reduction in the respiratory muscle strength and in the expansion of the thoracic complex. These reductions could interfere in the pulmonary volumes and consequently change hematosis and reduce tissue oxygenation [5].

Due to limitations imposed by CKD, studies suggest that physical activity is beneficial when incorporated into treatment [6]. This promotes improvements in cardiovascular and respiratory function, besides yielding positive effects on the quality of life of these patients [7–10].

Exercise protocols are usually developed in outpatient or in-hospital setting [11–13] with the most recent studies recommending that they should be performed during hemodialysis sessions [14–16]. Although improvement in physical fitness is expected in response to standardized aerobic activity, data correlating the changes obtained by a protocol that uses a cycle ergometer in respiratory function and functional capacity of patients undergoing hemodialysis are scarce.

The cycle ergometer is lightweight equipment, of low-cost and low-maintenance, that has received special attention in rehabilitation of patients with severe impairment of cardiac, respiratory, and muscular function [13]. Its use produces a positive response in increasing strength and cardiovascular conditioning assessed by testing submaximal [17–19]. The present study aimed to analyze the impact of cycle ergometer on respiratory function and functional capacity in patients with chronic kidney disease undergoing hemodialysis.

## 2. Sampling and Method

This is a randomized clinical study of adult patients with CKD undergoing hemodialysis, who were followed up at a renal unit in a quaternary hospital, following approval by the Research Ethics Committee of the same Institution (CAEE no. 41799915.5.0000.5479) and registered in the ClinicalTrials.gov database (NCT no. 02834026).

Patients aged 18 years or more, who had been undergoing hemodialysis for more than six months, were clinically stable, or had no pulmonary, musculoskeletal, or neurological disease, and who agreed to participate in the study by signing the informed consent form were included. Patients who needed urgent or elective surgical intervention during the protocol, those who had decompensation of prior heart disease with arrhythmia and/or precordial pain, ischemic cardiac event (< 3 months), significant valvular heart disease, or dysrhythmia, those using continuous and/or nighttime oxygen, or those who needed gait assistance devices or lower-limb orthoses were excluded.

All patients underwent the same protocol for hemodialysis during a four-hour period, three times a week, using polysulfone membrane capillaries (Fresenius) and standard bicarbonate dialysis solution (sodium: 138.0 mEq/L; potassium: 2.0 mEq/L; calcium: 2.5 mEq/L; magnesium: 1.0 mEq/L; chloride: 108.5 mEq/L; acetate: 3.0 mEq/L; bicarbonate: 32.0 mEq/L). The dose of agents stimulating erythropoiesis (ESA) and intravenous iron supplementation were free access throughout the study.

**Figure 1:**
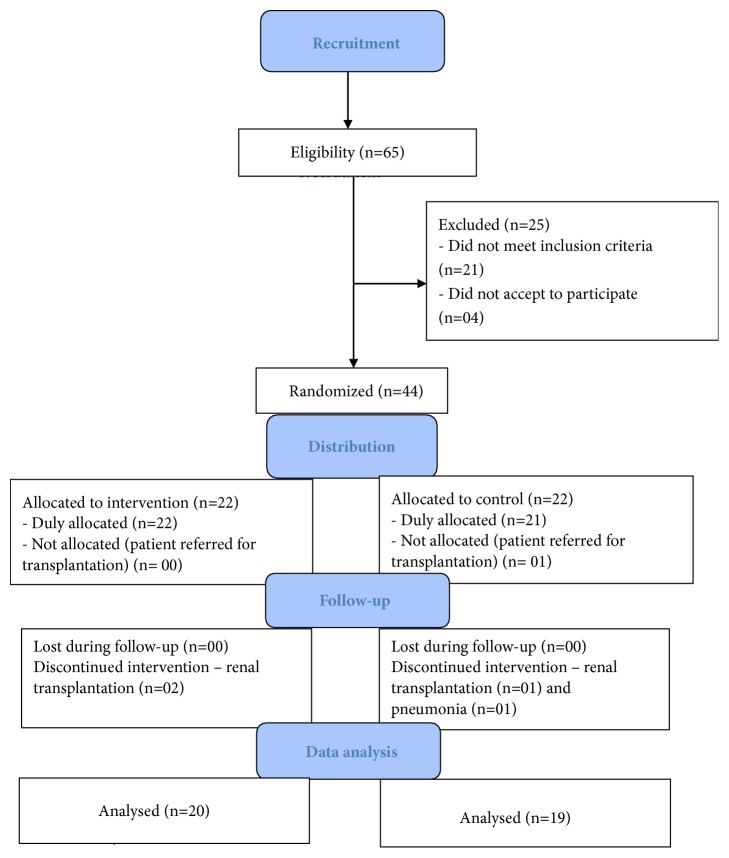
Flowchart representing recruitment, sample distribution, allocation, and data analysis.

The recruitment, allocation, and follow-up of patients participating in study progressed as shown in Figure 1 as suggested by the Consolidated Standards of Reporting Trials (CONSORT) [20].

Allocation concealment was ensured by using sealed and opaque envelopes, which contained a numerical randomization sequence as given by the software (java.util). Patients were randomized into two groups: treatment group (TG), with patients undergoing the protocol with the cycle ergometer, and control group (CG), with those who did not undergo the protocol.

During group allocation, patients in the CG were referred for renal transplantation. During data collection, in turn, two patients in the TG discontinued the intervention for renal transplantation. In the CG, the follow-up of two patients was discontinued, one due to kidney transplantation and the other due to pneumonia.

All patients were evaluated before and after the follow-up period. From medical records, the following data were collected: levels of hematocrit (Ht, %), hemoglobin (Hb, g/dL), creatinine (mg/dL), urea (mg/dL), serum albumin (g/dL), measure of hemodialysis adequacy (Kt/V), and hemodialysis treatment time (years).

Participants were assessed for height (m), weight (kg), body mass index (BMI, kg/m2), and tested for maximal respiratory pressures and peak flow, in spirometry. They also underwent the six-minute walk test (6MWT). All tests were conducted before the beginning of the hemodialysis first weekly session, so that the interval would not be greater than two interdialytic days.

The evaluator was blind for the allocation of patients to groups and study objectives and followed the standardization in order to carry out the entire data collection. Spirometry was performed using the Koko Spirometer apparatus from PDS Instrumentation, with values predicted by the equation described by Pereira et al. [21].

As they were tested, patients remained in the seated position and used a nasal clip. The following values were assessed: forced vital capacity (FVC), forced expiratory volume in the first second (FEV1), and peak expiratory flow (PEF).

Maximal inspiratory pressure (Pimax) and maximal expiratory pressure (Pemax) were measured using a manovacuometer (Comercial Médica), with measurements being taken with the patient seated, with their thorax and feet propped, while using a nasal clip.

First, the patient was instructed to hold the manovacuometer and tighten the mouthpiece firmly against the lips and then perform a maximal inspiration from the residual volume for measuring Pimax and a maximum expiration from the total lung capacity for determining Pemax.

Three measurements of Pimax and Pemax were taken with a resting time of 30 to 60 seconds between them, with the highest value obtained being used. Patients underwent a peak flow measurement test, conducted on a Peak Flow Meter (Assess) apparatus. Each patient was asked to maintain the orthostatic position, while using a nasal clip and after a maximum inspiration, in order to perform a forced expiration through the mouthpiece. Following the supervised training, three measurements were then taken and only the highest value found was considered, with variations no greater than 40 liters/minute (l/min) across measurements [22].

The 6MWT consists of a 6-minute free walk, as fast as possible, across a flat surface with 30 m length scaled at each meter in accordance with the recommendation from the American Thoracic Society (ATS) [19].

Before the start of the test, the following were measured: systolic and diastolic blood pressure (SBP and DBP, mmHg), as measured with a sphygmomanometer (Tycos), heart rate (HR, bpm) and respiratory rate (f, rpm) as observed for one minute with the aid of a stopwatch, peripheral oxygen saturation (SpO2, %) with a Nonin oximeter, and ratings of perceived exertion with the Borg scale [23].

The total distance walked is recorded at the end of the walk, just like that measured with SBP, DBP, HR, f, SpO_2_, and Borg scale.

### 2.1. Follow-Up in the Control Group

The CG was evaluated at baseline, and the renal unit guidelines were maintained until the reevaluation occurred two months after that. During their participation in the study, it is routine for patients to receive guidance from doctors and nurses on food, fistula care, personal hygiene, and infection prevention measures. They are encouraged to maintain their routine activities of daily living; i.e., they must carry on working, studying, and performing routine activities. Patients were further advised to walk and perform a self-assessment of their shortness of breath and fatigue; there was, however, no protocol containing guidelines for their physical activity routine.

### 2.2. Training Protocol

The TG attended 24 training sessions, 3 times a week, over 8 consecutive weeks during the intradialytic period. The training started one hour after the hemodialysis began and the patient was monitored with a frequency meter. A ten-minute warm-up routine was performed with active upper and lower limb exercises in the seated position, followed by a 30-minute ergometer activity cycle for the lower limbs (ACTE, Mini Bike) positioned in front of the patient's chair, and another 10 minutes of cool-down with gradual rotation decrease in the ergometer until HR and blood pressure returned to parameters close to the initial ones [18].

The movement performed on the cycle ergometer had the intensity required to keep HR between 50 and 70% of its maximum (HRmax). This was calculated by using the formula described by Karvonen et al. [24] (HRmax = 220 - age in years).

### 2.3. Statistical Analysis

The homogeneity of sex distribution between the groups was analyzed by using the chi-squared test. For numerical variables, the Shapiro-Wilk test was used. The Wilcoxon test was used to compare the evaluation and reevaluation moments in each group and between them. To compare whether there was a difference between the TG and the CG, we considered the delta value (final value at the follow-up - baseline value at the evaluation) and the ratio between the groups. The level of significance used for all analyses was 0.05.

## 3. Results

Patients in the training group (TG) (n = 20) had mean age of 44.25 (± 11.30) years and BMI of 22.75 (± 2.79) kg/m2. TG patients had been on hemodialysis support for a mean of 6.65 (± 4.70) years. The etiology of CKD: there was chronic hypertensive nephrosclerosis in 11 patients, diabetes mellitus in seven, and cystic kidney disease in two.

The analysis of the control group (CG) (n = 19) yielded a mean age of 42.63 (± 11.16) years and BMI of 22.75 (± 1.89) kg/m2. The mean duration time of hemodialysis treatment in the CG was 7.16 years (± 3.78). The etiology of CKD was hypertensive nephrosclerosis in 10 patients, diabetes mellitus in five, chronic glomerulonephritis in three, and cystic kidney disease in one.

There were 12 women in the TG and 10 in CG. 32 patients of the sample used throughout the study period erythropoietin to maintain the Hb target between 11 and 12g/dL without statistical difference between groups. There was homogeneity in comparison of demographic and HD characteristics, including age (p = 0.657), BMI (p = 1.000), sex (p = 0.215), and time on hemodialysis (p = 0.713), as well as the other variables evaluated between the TG and CG performed before the intervention.

Tables 1 and 2 present evolution intragroup considering follow-up, baseline and comparison between groups data for all patients, including spirometric values, maximal respiratory pressures, peak flow, and 6MWT for groups TG and CG (Table 1) and results for laboratory tests (Table 2).

## 4. Discussion

In the present study, patients with CKD performed aerobic activity on a cycle ergometer during hemodialysis sessions and had positive clinical progression in regard to respiratory and functional parameters. Studies had shown that restoring physical capacity and including rehabilitation in the treatment of these patients result in a number of benefits in addition to reducing the disease's negative impact on muscle tissue [6–8].

The results indicate such improvement in the sample analyzed in this study, with the additional benefit of the possibility of performing exercise while receiving hemodialysis a period of some hours of complete physical inactivity in most hemodialysis centers. This study showed that, instead of sitting in the same position for three or four hours, the patient undergoing hemodialysis can use the time to exercise, and benefit from it.

The mean age in TG and CG and the time on hemodialysis treatment indicate a need to stimulate the prescription of supervised physical exercise during sessions, given that the population of hemodialysis patients is usually still professionally active and may remain in treatment for many years before undergoing transplantation. Optimizing the respiratory and muscular function in these patients is relevant, and even more important if the condition of patients in the control group is considered: it progressed negatively during the two months of follow-up in this study.

According to Howden et al. [15], patients on hemodialysis are less active and less tolerant to exercise and suffer from noticeable muscle deconditioning. Complications in the respiratory system were reported as related to changes in volume status, plasma oncotic pressure, bone and mineral metabolism, with concomitant pulmonary edema, pleural effusion, sleep apnea, and hypoxia [2]. In this study, we found that respiratory muscle strength showed a better response after the proposed training protocol, which may interfere positively with respiratory changes.

Patients with CKD who receive dialysis treatment are subject to rapid changes in volume and biochemical composition of body fluids, which may adversely affect respiratory muscle function [1, 4, 5]. We did not study the body fluids composition in the sample evaluated in this study; however, we found that Kt/V did not exhibit a significant change during the follow-up period.

The improved FVC, FEV_1_, PEF, Pi_max_, Pe_max_, and peak flow after the intervention in patients in the treatment group indicated clinical progression. One possible explanation for this finding was that the systematic aerobic training regimen performed with the use of the cycle ergometer improved the recruitment of type I fibers in the lower-limb musculature and resulted in cardiac conditioning, which reflects on the respiratory function. This hypothesis should be further tested.

The functional capacity as assessed in the 6MWT in subjects was significantly better after the treatment, which corroborates the study by Heiwe et al. [25]. The authors verified that regular aerobic exercise lasting 30 minutes three times a week improves the aerobic capacity, blood pressure, muscle strength, and health-related quality of life. These benefits occur in adult patients with CKD stages II to V and in those receiving dialysis, as well as in adults following renal transplantation.

Aerobic exercise has been valued for its cardiorespiratory benefits, for reducing the risk of coronary heart disease and increasing maximal oxygen consumption. It also improves oxygen extraction, lowers blood pressure and glycated hemoglobin, and improves glucose tolerance and insulin sensitivity [6, 13, 26, 27].

In the sample, we could observe an improvement in functional capacity related to cardiac and respiratory function. This finding was relevant because patients with CKD have reduced cardiorespiratory fitness when compared to the general population and those patients with CKD who are sedentary and have an increased risk of mortality [3]. The study design has not allowed for conclusions regarding mortality, but we changed the status of the sample evaluated when participants went from sedentary to active lifestyles due to their doing standardized physical activity.

In comparing laboratory tests, we found that the intervention group showed changes in their Ht, creatinine, urea, and albumin levels. This might have led to a reduced inflammatory cascade. The reducing inflammation, oxidative stress, and endothelial dysfunction may decrease both the morbidity and mortality of chronic kidney disease patients. Aerobic exercising and resistance training promote reduction of inflammatory cytokines, microalbuminuria, and anemia related to chronic diseases [6, 8–10, 12].

Kt/V, which is an indicative parameter of the quality of the dialysis, did not change in our study, and this may suggest that there were no negative variations in the patients who underwent the protocol. In other words, the fact that the participants took physical exercise did not worsen the quality of dialysis, supporting the prescription of the safely systematized exercise during dialysis, as already reported in the literature [8, 25, 27]. In fact, the 2005 Guidelines of the National Kidney Foundation recommends that exercise should be one of the pillars in therapy for adults on dialysis, especially aimed at the attempt to control cardiovascular risk factors [25–27].

The frequency, intensity, and duration of training, as well as the proposed activity, must form a basis and become routine, as well as the exercise modality and adherence to the program. These parameters are essential when attempting to achieve a specific goal with exercise programs [8, 25]. The protocol proposed here included the cycle ergometer in the dialytic routine so that it could increase patients' aerobic function and functional capacity, using the frequency, intensity, and duration of exercise indicated in the literature [13–15].

The cycle ergometer used during the dialysis sessions resulted in improvement of Ht, although we did not obtain a significant increase in Hb levels, but there was an absolute increase in its serum levels. The hypothesis for this finding was that exercise is shown as an instrument for optimizing consumption capacity and the quality of the maximum oxygen values [13]. It also proves to be safe during the first two hours of dialysis [8, 25].

Storer et al. [26] showed that the practice of physical activity with cycle ergometer increases oxygen consumption, potency, quadriceps resistance time, and strength and improves fatigability. The improvement in fatigability was observed in this study with the increase in the walk distance on 6MWT and decrease in ratings of perceived exertion given by the Borg scale.

As a limitation to our findings, we emphasize that we did not evaluate the maximal VO2 of the patients that would allow a more sensitive analysis of aerobic capacity and the response to training with the cycloergometer [8, 13]. We also did not use scales that allowed the understanding of the role of physical activity to reach the autonomy or self-cohesion [28] of the patients, so we were unable to highlight the exclusive association of the exercise program with the variables studied.

## 5. Conclusion

The systematic training regimen with a cycle ergometer during hemodialysis resulted in benefits in the respiratory function and functional capacity in patients with chronic kidney disease.

## Figures and Tables

**Table 1:** Respiratory function and functional capacity in the treatment group (TG, n = 20) and the control group (CG, n = 19) with intragroup comparison and between groups.

Variables	Groups	Baseline Mean (SD)	Follow-up Mean (SD)	*p* intragroup	*p* between groups
FCV (I)	TG	2.47 (0.78)	2.61 (0.78)	**0.003**	**0.005**
	CG	2.59 (0.87)	2.54 (0.81)	0.379	

FEV_1_ (l)	TG	1.88 (0.66)	2.08 (0.49)	**0.008**	**0.002**
	CG	2.01 (0.61)	1.91 (0.54)	0.110	

PEF (l/min)	TG	3.81 (1.44)	4.04 (1.40)	**0.006**	**0.001**
	CG	3.6 (1.12)	3.38 (0.93)	0.091	

Pi_max_ (cm/H_2_O)	TG	51.35 (10.22)	63.05 (17.53)	**0.001**	**0.001**
	CG	52.63 (17.30)	48.79 (16.85)	0.119	

Pe_max_ (cm/H_2_O)	TG	63.3 (16.22)	78.15 (9.77)	**0.001**	**0.001**
	CG	63.47 (17.64)	61.64 (18.95)	0.229	

Peak flow (l/min)	TG	292.3 (65.01)	313.90 (64.28)	**0.001**	**0.002**
	CG	290.21 (68.32)	274.21 (69.73)	**0.008**	

SBP (mmHg)	TG	139 (16.59)	140.50 (11.90)	0.532	0.738
	CG	140.53 (15.08)	143.16 (16.68)	0.287	

DBP (mmHg)	TG	82.5 (11.18)	86.00 (6.60)	0.115	0.864
	CG	83.42 (9.14)	86.32 (12.52)	0.322	

HR (bpm)	TG	96.35 (8.73)	96.50 (13.04)	0.963	**0.031**
	CG	97.05 (12.44)	106.00 (14.30)	**0.001**	

f (rpm)	TG	22.4 (2.66)	20.50 (4.22)	**0.05**	0.051
	CG	22.42 (3.49)	22.95 (3.42)	0.505	

SpO_2_ (%)	TG	96.95 (1.23)	97.50 (0.95)	0.077	0.243
	CG	96.16 (1.30)	96.26 (0.99)	0.65	

Borg	TG	11.3 (2.30)	10.90 (2.46)	0.504	**0.016**
	CG	11.37 (2.83)	13.37 (2.71)	**0.016**	

Distance (m)	TG	348.65 (96.52)	386.90 (19.38)	**0.001**	**0.001**
	CG	327.16 (62.86)	325.00 (59.80)	0.257	

FVC: forced vital capacity; FEV_1_: final expiratory volume in the first second; PEF: peak expiratory flow; Pi_max_: maximal inspiratory pressure; Pe_max_: maximal expiratory pressure; SBP: systolic blood pressure; DBP: diastolic blood pressure; HR: heart rate; f: respiratory frequency; SpO_2_: peripheral oxygen saturation; SD: standard deviation.

**Table 2:** Laboratory tests results in the treatment group (TG, n = 20) and the control group (CG, n = 19), with intragroup comparison and between groups.

Variables	Groups	Baseline Mean (SD)	Follow-up Mean (SD)	*p *intragroup	*p *between groups
Ht (%)	TG	32.54 (4.44)	33.32 (4.05)	0.176	**0.008**
	CG	33.96 (5.97)	31.75 (5.42)	**0.028**	

Hb (g/dL)	TG	10.28 (1.37)	10.70 (1.14)	0.191	**0.037**
	CG	11.06 (2.23)	10.25 (1.66)	0.111	

Creatinine (mg/dL)	TG	10.98 (1.44)	10.36 (1.69)	**0.016**	**0.005**
	CG	10.08 (1.68)	10.32 (1.66)	0.172	

Urea (mg/dL)	TG	149.47 (31.87)	142.44 (28.68)	**0.029**	**0.032**
	CG	144.1 (32.02)	147.90 (28.05)	0.337	

Kt/V	TG	1.39 (0.19)	1.36 (0.185)	0.532	0.236
	CG	1.41 (0.25)	1.48 (0.19)	0.327	

Albumin (g/dL)	TG	3.53 (0.37)	3.79 (0.25)	**0.001**	**0.001**
	CG	3.54 (0.40)	3.46 (0.41)	0.29	

Ht: hematocrit; Hb: hemoglobin; Kt/V: measure of hemodialysis adequacy; SD = standard deviation.

## Data Availability

No data were used to support this study.
